# Altered dynamics of broad-leaved tree species in a Chinese subtropical montane mixed forest: the role of an anomalous extreme 2008 ice storm episode

**DOI:** 10.1002/ece3.1433

**Published:** 2015-03-06

**Authors:** Jielin Ge, Gaoming Xiong, Zhixian Wang, Mi Zhang, Changming Zhao, Guozhen Shen, Wenting Xu, Zongqiang Xie

**Affiliations:** 1 State Key Laboratory of Vegetation and Environmental Change, Institute of Botany, Chinese Academy of SciencesNo.20 Nanxincun, Xiangshan, Beijing, 100093, China; 2 University of Chinese Academy of SciencesBeijing, 100049, China; 3 Administration Bureau of Shennongjia National Nature ReserveShennongjia, Hubei, 442421, China; 4 Northwest Institute of Plateau Biology, Chinese Academy of SciencesXining, 810008, China

**Keywords:** Diameter growth rate, frost damage, leaf habit, recruitment and mortality, the extreme climatic event

## Abstract

Extreme climatic events can trigger gradual or abrupt shifts in forest ecosystems via the reduction or elimination of foundation species. However, the impacts of these events on foundation species' demography and forest dynamics remain poorly understood. Here we quantified dynamics for both evergreen and deciduous broad-leaved species groups, utilizing a monitoring permanent plot in a subtropical montane mixed forest in central China from 2001 to 2010 with particular relevance to the anomalous 2008 ice storm episode. We found that both species groups showed limited floristic alterations over the study period. For each species group, size distribution of dead individuals approximated a roughly irregular and flat shape prior to the ice storm and resembled an inverse J-shaped distribution after the ice storm. Furthermore, patterns of mortality and recruitment displayed disequilibrium behaviors with mortality exceeding recruitment for both species groups following the ice storm. Deciduous broad-leaved species group accelerated overall diameter growth, but the ice storm reduced evergreen small-sized diameter growth. We concluded that evergreen broad-leaved species were more susceptible to ice storms than deciduous broad-leaved species, and ice storm events, which may become more frequent with climate change, might potentially threaten the perpetuity of evergreen-dominated broad-leaved forests in this subtropical region in the long term. These results underscore the importance of long-term monitoring that is indispensible to elucidate causal links between forest dynamics and climatic perturbations.

## Introduction

Forest ecosystems are dynamic, and alterations occur continuously at the individual, population, and community level due to an imbalance between mortality, recruitment, and growth (Condit et al. 1992; Zhou et al. 2014). Long-term monitoring of forest ecosystems worldwide has shown that forests are undergoing directional shifts in composition and structure and becoming more dynamic during the last decades (Laurance et al. 2004; Lewis et al. 2009; Bulafu et al. 2013; Luo and Chen 2013). Current environmental changes, such as drought, warming, and rising atmospheric carbon dioxide concentrations, have been identified as main drivers that could potentially modify forest structure and function such as carbon sequestration (Allen et al. 2010; Enquist and Enquist 2011; Peng et al. 2011; Zhou et al. 2014). However, a full understanding of underlying causes and consequences of these dynamic shifts is still far from being reached (Lewis et al. 2009).

Growing evidence has accumulated on the roles of extreme climatic events on forest dynamics over the last years (Lloret et al. 2012; Reichstein et al. 2013). Ice storms representing an important form of extreme climatic event often contribute to shifts in structure, composition, and even patterns of forest vegetation (Weeks et al. 2009; Zhou et al. 2011; Lind et al. 2014). While ice storms may promote establishment and survival of shade-intolerant species by creating light gaps and modifications to the microenvironment near the ground and thus retard forest succession, they may also accelerate forest development when there is abundant advance regeneration of other preexisting species under a shade-intolerant canopy (Lafon 2004; Takahashi et al. 2007; Holzmueller et al. 2012). Either scenario could occur, depending largely upon the nature of ice storm such as occurrence, frequency, or intensity, as well as initial stand condition, that is, stand age, stem density (Duguay et al. 2001; Lloret et al. 2012). There is, however, a paucity of studies considering ice storms' effect on forest dynamics.

Previous studies examining the effects of ice storms on forest ecosystems have mainly focused on responses of trees following snow damages and greatly advanced our understanding of the role of ice storms in forest dynamics (Rhoads et al. 2002; Man et al. 2011; Shi et al. 2013). For example, some studies have reported the forms and differences of damage among species using arbitrarily chosen damage classes (Irland 2000; Hopkin et al. 2003; Man et al. 2011; Shao et al. 2011); others have documented tree-ring growth, mortality, and ecophysiological responses of trees after ice storms (Boyce et al. 2003; Smith and Shortle 2003; He et al. 2011b). However, these published studies are mainly based on temporary observation of ice storm-damaged forests, and they may miss detailed demographic processes such as growth, recruitment, and mortality (Condit et al. 1992; Weeks et al. 2009). Therefore, it is recommended to carry out long-term monitoring via permanent plots to capture these demographic data to understand how ice storms driver forest dynamics (Condit et al. 1992; Duguay et al. 2001). However, to date, few studies have addressed the effects of ice storms on forest dynamics based on established permanent forest inventory records (Weeks et al. 2009).

Generally, ice storms are recurring events in temperate and boreal regions worldwide but rarely occur in subtropical and tropical regions (Takahashi et al. 2007; Zhou et al. 2011; Reichstein et al. 2013). However, a broad band of subtropical China from 10 January to 6 February in 2008 experienced an unprecedented catastrophic ice storm with a record-setting duration and severity in five decades, opening a unique window of opportunity to understand the impacts of ice storms on forest dynamics of this region (Zhou et al. 2011; Du et al. 2012).

The mixed montane evergreen and deciduous broad-leaved forest is the climax vegetation in the northern subtropical zone in central China. Records on dynamics of this montane forest are still underrepresented despite well-understood floristic characteristics (Zhang et al. 2003)**.** Today, this forest is becoming one of the most threatened ecosystems worldwide and potentially vulnerable to climate change (Zhang et al. 2003). Earlier work has implied that short-term winter temperature extremes might be critical in controlling over broad-leaved tree species survival and thus influence forest dynamics (Harrison et al. 2010; Kollas et al. 2014). Moreover, evergreen and deciduous broad-leaved species, the two foundation components, occupy distinct vertical strata in this forest and thus might respond differently to this long-duration freezing event (Zhang et al. 2003; Butt et al. 2014). Consequently, precise and quantitative analyses on tree demography of these two main species groups will provide important insights on the effects of ice storm on forest dynamics and vulnerability of this forest to such climatic perturbation under future climate change.

In this study, we took advantage of the pre- and postice storm surveys based on a permanent plot to assess the effects of the 2008 ice storm on forest dynamics. We hypothesized that divergent leaf habit (evergreen and deciduous) influenced each other's demographic performance during the ice storm period. Specifically, we addressed the following questions: (1) What were the demographic characteristics of both evergreen and deciduous broad-leaved species and how did these change after the ice storm? (2) Were evergreen broad-leaved species more susceptible to ice storm than deciduous broad-leaved species?

## Materials and Methods

### Study site

We performed this study in a montane mixed evergreen and deciduous broad-leaved forest, located on the southern slope of the Shennongjia region (31°19′4″ N, 110°29′44′' E) listed among the 34 global biodiversity hot spots (Myers et al. 2000). The region falls in a climate and vegetation transition between the subtropical and warm temperate climatic zone. The climax vegetation is the montane mixed evergreen deciduous broad-leaved forest in this region. Mean annual precipitation is ca. 1350 mm, and the mean annual air temperature is ca. 10.6°C. Records from the nearest meteorological station, National Field Research Station for Forest Ecosystem in Shennongjia (31°19′22″N, 110°29′06″E), over the study period (2001–2010), revealed no directional trend in total annual rainfall and air temperature. In early 2008, however, an intense ice storm swept across this region and last up to 28 days with a minimum air temperature of −17.6°C, significantly 4°C lower than daily minimum air temperature. Precipitation, falling mostly as freezing rain during the ice storms, was 30% greater and the number of snowing days more than doubled in any previous years (Li et al. 2009).

### Data collection

In 2001, we laid out a 120 m × 80 m permanent plot within a *Fagus engleriana – Cyclobalanopsis multinervis* mixed forest patch, representing a climax forest. The elevation range between the highest and lowest points within the plot was 125 m. The topography of this plot is very steep, with an average slope of 30°. Data collection followed the standard census protocol employed worldwide (Condit et al. 2014). All free-standing woody stems with diameter at breast height equal to or greater than 4 cm were individually tagged with a numbered aluminum tag, identified to species, measured for dbh, and mapped using Cartesian coordinates. We undertook re-censuses in 2006 and 2010. We also noted dead woody individuals and recorded newly recruited trees that met the measurable size criteria in the re-censuses.

### Data analysis

We classified recorded species (excluding coniferous species, accounting for ca. 1%) into two species groups: evergreen and deciduous broad-leaved species. This classification was principally based on the checklist of plants of this region and the Flora of China (The Editorial Board of Flora of China 2004).

To describe the compositional shift in this forest, we used Bray–Curtis dissimilarity index by calculating the relative dominance ratio for each species. The relative dominance ratio (RDR) of each species was calculated as RDR = (relative stem density + relative basal area)/2 (Takahashi et al. 2007; Bulafu et al. 2013). The Bray–Curtis dissimilarity index is bound between 0 and 1, where 1 means species composition has changed completely between time intervals, and 0 means no shift in species composition over time (Clarke et al. 2006).

To evaluate demographic process in this forest, we utilized the pre- and postice storm survey data to calculate mortality (m) and recruitment rates (r) for each species group by following logarithmic models as previously described: m = (ln(N_0_) – ln(Ns))/t × 100,*r* = (ln (Nt) – ln(Ns))/t × 100, where Nt and N_0_ represent the size (stem number or basal area) of specific species group at time t and time 0, respectively, and Ns represents the size of survivors at time t (Laurance et al. 2004; Condit et al. 2014).

We took Kolmogorov–Smirnov two-sample goodness-of-fit tests to analyze size distribution of dead individuals for each species group (Takahashi et al. 2007; Enquist and Enquist 2011). We also determined mean annualized diameter growth rate for each species group by difference in diameter between two consecutive periods dividing by the number of years. We examined differences in mean annual diameter growth rates between both species groups using Mann–Whitney *U*-tests while differences between two census intervals for each species group were performed by paired *t*-test (Laurance et al. 2004; Zhou et al. 2014). We conveyed all statistical analysis by R free software (R Core Team 2011) following the guidance (Condit et al. 2014).

## Results

### Diversity and floristic composition

Deciduous broad-leaved species dominated in the upper-canopy strata and evergreen broad-leaved species in the subcanopy layer within the forest vertical profile over the study period. Most of the species richness for woody species (ca. 75%) pertained to deciduous broad-leaved species, and total species richness of evergreen and deciduous broad-leaved species fluctuated little before and after the ice storm (Table1). Deciduous broad-leaved species group, mainly consisting of *Fagus engleriana* and *Dendrobenthamia japonic*a, contributed 46.3% of stem density and 67.2% of basal area, while evergreen broad-leaved species predominately *Cyclobalanopsis multinervis* and *Rhododendron hypoglaucum* accounted for 52.7% of stem density and 31.5% of basal area in 2001 (Table2). Deciduous broad-leaved species had greater basal area but lower stem density than evergreen broad-leaved species throughout the study census period (Table1). Stem density and basal area for both species groups increased prior to the ice storm but declined slightly after the ice storm, with the exception that basal area of deciduous broad-leaved species increased continuously. Deciduous broad-leaved species maintained a larger mean stem diameter than evergreen broad-leaved species and both species groups increased continuously mean stem diameter across the study period (Table1). Although Bray–Curtis dissimilarity index for each species group increased after the ice storm, the absolute value of this index was still small (Table3). Therefore, we assume that species composition for each species group was relatively stable between 2001 and 2010.

**Table 1:** Structural characteristics of the two species groups during the study period (2001–2010)

Species group	Species richness			Stem density (stems ha^−1^)			Basal area (m^2^ ha^−1^)			Mean stem diameter (cm)		
	2001	2006	2010	2001	2006	2010	2001	2006	2010	2001	2006	2010
Evergreen species	20	21	21	1406	1475	1383	12.70	13.76	13.16	9.24	9.41	9.5
Deciduous species	60	62	61	1260	1281	1194	26.52	28.25	28.65	13.9	14.18	14.8

**Table 2:** Species characteristics of the most abundant woody species

Species	Group	Stem density (stems ha^−1^)			Basal area (m^2^ ha^−1^)		
		2001	2006	2010	2001	2006	2010
*Cyclobalanopsis multinervis*	E	880	926	848	7.84	8.56	8.02
*Rhododendron hypoglaucum*	E	219	227	214	1.14	1.27	1.29
*Lithocarpus henryi*	E	81	83	68	2.10	2.21	1.98
*Ilex pernyi*	E	53	58	68	0.16	0.19	0.22
*Quercus engleriana*	E	39	38	38	0.42	0.43	0.45
*Lyonia ovalifolia*	E	23	24	28	0.15	0.16	0.23
*Fagus engleriana*	D	272	275	264	5.56	5.83	5.93
*Dendrobenthamia japonica*	D	121	126	122	0.85	0.92	0.92
*Acer pictum subsp. mono*	D	73	72	60	1.65	1.73	1.69
*Corylopsis platypetala*	D	52	56	47	0.13	0.15	0.13
*Acer griseum*	D	52	53	53	1.15	1.25	1.32
*Lindera obtusiloba*	D	49	48	46	0.66	0.71	0.74

E: Evergreen broad-leaved species; D: Deciduous broad-leaved species.

**Table 3:** Bray–Curtis dissimilarity index of the relative dominance ratio for each species group

Species group	2001–2006	2006–2010	2001–2010
Evergreen species	0.012	0.029	0.028
Deciduous species	0.019	0.029	0.036

### Recruitment and mortality

We detected a large range of variation in mortality and recruitment based on stem number and basal area for each species group before and after the ice storm (Table4). In both census intervals, most of the recruited stems belonged to evergreen broad-leaved species. Stem recruitment rate of evergreen broad-leaved species was nearly the double of deciduous broad-leaved species prior to the ice storm but an opposite pattern was displayed after the ice storm. Moreover, evergreen broad-leaved species increased stem number recruitment rate while deciduous broad-leaved species decreased through the whole period.

**Table 4:** Recruitment and mortality rate for each species group

Species Group	Based on stem number				Basal on basal area			
	Recruitment rate (% yr^−1^)		Mortality rate (% yr^−1^)		Recruitment rate (% yr^−1^)		Mortality rate (% yr^−1^)	
	2001–2006	2006–2010	2001–2006	2006–2010	2001–2006	2006–2010	2001–2006	2006–2010
Evergreen species	1.18	0.19	0.22	1.79	1.79	2.12	0.17	3.24
Deciduous species	0.68	1.09	0.35	2.86	1.62	1.79	0.36	1.44

Overall, both species groups presented relatively low mortality rates based on stem number (Table4). Deciduous broad-leaved species showed higher stem mortality rate than evergreen broad-leaved species over the study period. Therefore, both species groups showed positive population growth before the ice storm and negative growth after the ice storm. Basal area mortality rate was lower than basal area recruitment rate on both occasions for both species groups, except that evergreen broad-leaved species displayed the highest negative balance (−1.12%) after the ice storm because of basal area mortality rate surpassing basal area recruitment rate.

**Figure 1:**
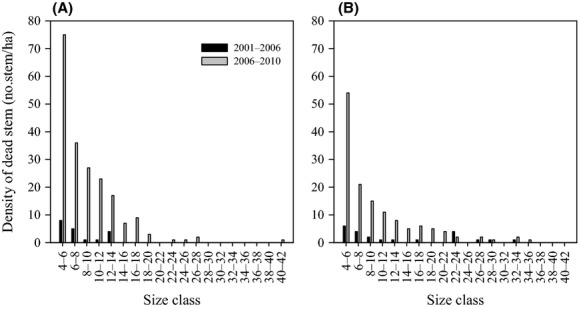
Size distributions of dead individuals for each species group during the study period (2001–2010). (A) Evergreen broad-leaved species; (B) Deciduous broad-leaved species.

Besides, size distribution of dead individuals differed definitely before and after ice storm for both species groups (*P* < 0.05) (Fig.1). Specifically, size distribution of dead individuals for each species group showed an irregular mortality distribution prior to the ice storm but approached an inverted J mortality distribution after the ice storm with a higher number of dead individuals in smaller size classes.

### Diameter growth rate

**Figure 2:**
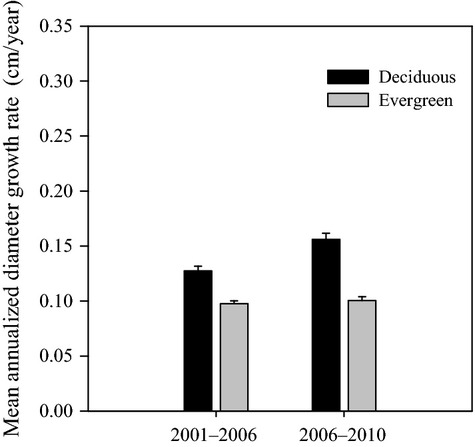
Mean diameter growth rate for each species group.

**Figure 3:**
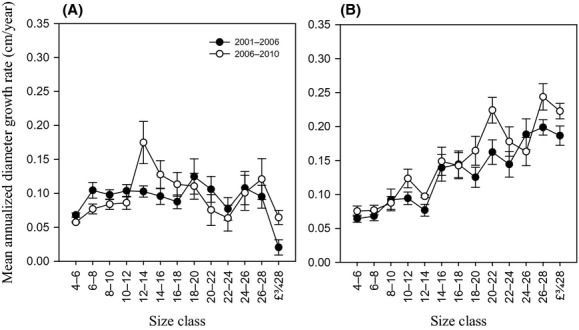
Relationships between mean diameter growth rate and size for each species group during the study period (2001–2010). (A) Evergreen broad-leaved species; (B) Deciduous broad-leaved species.

Diameter growth rate of surviving individuals over both census intervals varied greatly on species group and size class. Evergreen broad-leaved species registered significantly lower mean diameter growth rate than deciduous broad-leaved species on both census intervals (Mann–Whitney *U*-tests, *P* < 0.05) (Fig.2). Evergreen broad-leaved species did not change its overall diameter growth while deciduous broad-leaved species increased its overall diameter growth after the ice storm (Fig.2). Both specie groups showed size-dependent diameter growth rate over time: evergreen broad-leaved species expressed a hump-shaped diameter growth with a peak at intermediate diameters (12–14 cm); diameter growth rate for deciduous broad-leaved species increased monotonically with size. The preice storm diameter growth rate was higher than postice storm diameter growth rate for evergreen small size classes (4–12 cm) (paired *t*-test, *P* < 0.05) (Fig.3).

## Discussion

### Compositional stability for each species group despite the ice storm

The species composition and structure in this forest typify montane mixed forests in this region (Wu 1980). Evergreen broad-leaved species exhibited greater stem density but less basal area and species richness than deciduous broad-leaved species, which may be a representative characteristic of this forest. The composition and structure of forest ecosystems may often shift dramatically after extreme climatic events (Holzmueller et al. 2012; Lloret et al. 2012). However, we found both species groups experienced limited compositional shifts in the short term despite the atypical climate. Our result parallels that of these studies in the temperate deciduous broad-leaved forests (Steven et al. 1991; Takahashi et al. 2007) but contradicts with the finding in the evergreen broad-leaved forests (He et al. 2011b; Man et al. 2011). Takahashi et al. (2007) have found a severe ice storm substantially decreased stand basal area and stem density but did not alter the overall species composition in a temperate deciduous broad-leaved forest, while Man et al. (2011) demonstrated that the ice storm-induced directional shifts in forest composition due to species-specific differences of damage in a subtropical evergreen broad-leaved forest. The differences of these forests in response to ice storms may indicate that we could not generalize conclusions on the impact of ice storms on forest structure considering discrepancies in stand conditions and variation in intensity of ice storms within and among forest types.

Our result could be reasonably explained by the existence of stabilizing processes, which minimized the effects of the 2008 ice storm on this forest (Lloret et al. 2012). First, in our study, no dominant tree species populations collapsed through the whole study period, though high variable. Second, mortality rate of both species groups was too low to initiate dramatic vegetation shifts (Acker et al. 2015). Moreover, faster diameter growth of persistent individuals and new postice storm recruitment for each species group to some degree balanced the increased loss induced by mortality related to ice storm. Additionally, frequently exposed to conspicuous frosts in winter or early spring, woody species in this region may produce ecological stress memory and improved tolerance of abrupt ice storms (Reichstein et al. 2013; Walter et al. 2013). There is a possibility that our time window of field monitoring may be relatively insufficient for detecting significant changes in floristic composition of long-lived trees species in this forest. Therefore, it will be interesting to find out whether compositions for both species groups maintain limited stability over longer periods (Condit et al. 1992).

### Altered demographic patterns for each species group

We found that there were some imbalances between mortality and recruitment rate based on either stem number or basal area for each species group, though highly variable. In particular, mortality and recruitment rates underwent substantial alterations from pre-2006 to the 2010 census, where mortality rates began to exceed recruitment, reversing earlier patterns. Our result is consistent with earlier studies (Lloret et al. 2012), especially relating to evergreen broad-leaved trees, which showed negative population growth. Several explanations perhaps accounted for the variation in demographic rates over time in this forest. The most plausible and prevalent cause was the 2008 ice storm. Both species groups likely sustained great loss in the canopy stratum and led to small-sized individual death when ice storm occurred. Moreover, evergreen broad-leaved species presented more overwinter foliages, providing more beneficial interface for ice accumulation (He et al. 2011b), and maintained high leaf photosynthetic capacity concurrently with low freezing resistance, suffered more physical and physiological damage from the ice storm than the deciduous (Nadrowski et al. 2014; Rehm et al. 2014). Other studies in the evergreen broadleaved forests confirmed our explanation, which indicated that the deciduous were less susceptible to breakage following the ice storm (He et al. 2011b; Nadrowski et al. 2014). Furthermore, ice storm-damaged canopies induced low recruitment of some species via reduced flowering opportunities, seed production (Du et al. 2012; Zhou et al. 2013; Allen et al. 2014). Besides, it may have resulted from temporal discontinuity with mortality occurring first and thus releasing resource for recruitments of both species groups (Lloret et al. 2012).

The differences in recruitment between both species groups in both census intervals indicated that the ice storm might restrict recruitment for evergreen broad-leaved species but improve conditions for preexisting small-sized deciduous broad-leaved individuals that have been inhibited by low light in the understory prior to the ice storm. Previous studies in the evergreen broad-leaved forests supported our results (He et al. 2011b; Nadrowski et al. 2014). We argued that biophysical and physiological differences of understory saplings between the evergreen and the deciduous mainly contributed to this discrepancy. In this mixed forest, upper-canopy deciduous broad-leaved species could shelter lower-statured evergreen broad-leaved species. Ice storm-induced damage to upper-canopy deciduous broad-leaved trees led to less hospitable microenvironment for the evergreen, incurring death of subcanopy evergreen individuals (Xiong et al. 2002; Zhang et al. 2003; Butt et al. 2014). Alternatively, deciduous broad-leaved species are less shade-tolerant and could achieve faster growth in small-sized individuals because of additional light availability due to canopy openness created by the ice storm and hence facilitate their recruitments (Man et al. 2011; Du et al. 2012). Moreover, deciduous broad-leaved species showed higher freezing tolerance in terms of tree organs such as twigs, stems, and buds than the evergreen, thus led to their greater resistance to ice storm-induced physiological damages (Harrison et al. 2010; Rehm et al. 2014). Finally, ice storms could initiate larger numbers of deciduous species' new sprouts from dormant and adventitious buds and increase growth rate among residual saplings, favoring their post-ice storm recruitments (Zhang et al. 2003; Beaudet et al. 2007; Weeks et al. 2009).

### Faster growth for the deciduous and slower growth for evergreen small-sized individuals

Deciduous species groups increased mean diameter growth after the ice storm. This result contrasts with earlier finding in some forests (Wright et al. 2010), but is in line with some recent observations in other forests (Tanner et al. 2014). Our finding was surprising because ice storm likely reduced tree vigor as evident from higher postice storm mortality. Nevertheless, several ideas could account for this phenomenon. We speculated that higher postice storm mortality could also explain our observation pattern to some degree. The elevated growth was probably linked to reduced competition and increased amount of sun exposure on residual stems, inflicted by the decreased vigor of canopy related to the ice storm. Reduced stem density and basal area stimulated growth of deciduous survivors owing to additional resource availability such as nutrients, light, and growing space belowground and aboveground, as the symmetric and/or asymmetric competition released (Weeks et al. 2009; Luo and Chen 2013). Furthermore, the 2008 ice storm perhaps increased the size and prevalence of canopy gaps and dramatically altered vertical and horizontal gradient of light (Zhou et al. 2011; Shi et al. 2013). Under these conditions, an increase in light and an elevated frequency and size of sun flecks ameliorated the microenvironment beneath the canopy layer (Xiong et al. 2002). Consequently, additional residual resources, that is, nutrients, generated after the ice storm, may be channeled to benefit the growth of these deciduous individuals (Duguay et al. 2001). Another possible explanation was that deciduous tree architectures were altered by epicormic growth caused by the removal of dominance by the ice storm (Beaudet et al. 2007). Ice storm removed tree apical meristems, favored the lateral expansion of overstory tree crowns (Gu et al. 2008), and incurred more larger photosynthetic areas available for assimilation and better access to light, which may benefit stem growth for the deciduous (Butt et al. 2014).

Furthermore, we found that the ice storm perhaps lowered the growth rate of evergreen small-sized individuals. We speculated that due to secondary damage caused by surrounding damaged trees and physiological injuries such as xylem embolism and cambium browning, ice storms destroyed expensive, understory long-lived evergreen leaves, representing substantial loss of potential carbon assimilation, whereas deciduous trees generally have no leaves when ice storms occurred (Irland 2000; Kollas et al. 2014; Rehm et al. 2014). Moreover, damage of the root system may be another plausible cause of this observed pattern. Ice storms in 2008 commonly caused uprooting in understory small-sized trees due to shallow root system, and effectively reduced tree height to ground level and exposed roots, limiting the ability to access above- and below-ground resources (Nadrowski et al. 2014). Tree survival after uprooting may rely upon shade tolerance and capacity to access belowground resources (Nadrowski et al. 2014). Evergreen species in the understory were usually more shade-tolerant and thus more insensitive to ice storm-caused increased canopy light penetration than the deciduous (Xiong et al. 2002). Therefore, in the recovery process of the postice storm, these damaged small-sized evergreen trees responded with a subsequent prioritization of root and crown foliage production at the expense of stem growth (Duguay et al. 2001; Gu et al. 2008; Zhou et al. 2011). Recent work performed after the same ice storm in an evergreen broad-leaved forest confirmed our explanation (Man et al. 2011). Our result also explained the aforementioned lower recruitment rate for evergreen broad-leaved species following the ice storm. Therefore, we argued that evergreen broad-leaved species were more susceptible to the ice storm in 2008 than deciduous broad-leaved species.

## Conclusions

The results from this study suggest that ice storms may have significant implications for subtropical broad-leaved forests. Our opportunistic study of the extreme climatic event tentatively indicates that in spite of the severe ice storm, both species groups persisted with limited alterations in floristic composition during the study period, together maintaining the montane mixed evergreen and deciduous broad-leaved forest physiognomy. However, recent associated studies in other forests have revealed that this kind of ice storms has destroyed forest composition (Man et al. 2011; Zhou et al. 2011, 2013). These conflicting conclusions have highlighted the need for additional case studies to enhance our understanding the effects of ice storms regarding rarity and unpredictability of their occurrence. Furthermore, another important implication of our work is that the uneven impacts of the ice storm on tree demography between different foundation species groups. Our results indicate that the evergreen was more vulnerable to ice storms than the deciduous. We speculate ice storms possibly provided a competitive advantage for the deciduous at the cost of the evergreen in terms of altered patterns of tree regeneration (including recruitment and small-sized growth). Therefore, as demonstrated by other comparable results (He et al. 2011a; Man et al. 2011; Shi et al. 2013), we argue that ice storms might potentially threaten the perpetuity of evergreen-dominated broad-leaved forests in this subtropical region with increasing frequency of ice storms (Duan et al. 2012; Zhou et al. 2013). However, in view of unpredictability of extreme climatic events like ice storms under future climate change scenario (Reichstein et al. 2013; Zhou et al. 2013), long-term forest monitoring is indispensible to further elucidate causal links between forest dynamics and climatic perturbations.

## References

[b1] Acker SA, Boetsch JR, Bivin M, Whiteaker L, Cole C, Philippi T (2015). Recent tree mortality and recruitment in mature and old-growth forests in western Washington. Forest Ecol. Manag.

[b2] Allen CD, Macalady AK, Chenchouni H, Bachelet D, McDowell N, Vennetier M (2010). A global overview of drought and heat-induced tree mortality reveals emerging climate change risks for forests. Forest Ecol. Manag.

[b3] Allen RB, Hurst JM, Portier J, Richardson SJ (2014). Elevation-dependent responses of tree mast seeding to climate change over 45 years. Ecol. Evol.

[b4] Beaudet M, Brisson J, Gravel D, Messier C (2007). Effect of a major canopy disturbance on the coexistence of *Acer saccharum* and *Fagus grandifolia* in the understorey of an old-growth forest. J. Ecol.

[b5] Boyce RL, Friedland AJ, Vostral C, Perkins TD (2003). Effects of a major ice storm on the foliage of four New England conifers. Ecoscience.

[b6] Bulafu C, Baranga D, Mucunguzi P, Telford R, Vandvik V (2013). Massive structural and compositional changes over two decades in forest fragments near Kampala, Uganda. Ecol. Evol.

[b7] Butt N, Bebber DP, Riutta T, Crockatt M, Morecroft MD, Malhi Y (2014). Relationships between tree growth and weather extremes: spatial and interspecific comparisons in a temperate broadleaf forest. Forest Ecol. Manag.

[b8] Clarke KR, Somerfield PJ, Chapman MG (2006). On resemblance measures for ecological studies, including taxonomic dissimilarities and a zero-adjusted Bray-Curtis coefficient for denuded assemblages. J. Exp. Mar. Biol. Ecol.

[b9] Condit R, Hubbell SP, Foster RB (1992). Short-term dynamics of a neotropical forest. Bioscience.

[b10] Condit R, Lao S, Singh A, Esufali S, Dolins S (2014). Data and database standards for permanent forest plots in a global network. Forest Ecol. Manag.

[b11] Du Y, Mi X, Liu X, Ma K (2012). The effects of ice storm on seed rain and seed limitation in an evergreen broad-leaved forest in east China. Acta Oecol.

[b12] Duan J, Zhang QB, Lv L, Zhang C (2012). Regional-scale winter-spring temperature variability and chilling damage dynamics over the past two centuries in southeastern China. Clim. Dynam.

[b13] Duguay SM, Arii K, Hooper M, Lechowicz MJ (2001). Ice storm damage and early recovery in an old-growth forest. Environ. Monit. Assess.

[b14] Enquist BJ, Enquist CAF (2011). Long-term change within a Neotropical forest: assessing differential functional and floristic responses to disturbance and drought. Global Change Biol.

[b15] Gu L, Hanson PJ, Post WM, Kaiser DP, Yang B, Nemani R (2008). The 2007 Eastern US spring freeze: increased cold damage in a warming world?. Bioscience.

[b16] Harrison SP, Prentice IC, Barboni D, Kohfeld KE, Ni J, Sutra JP (2010). Ecophysiological and bioclimatic foundations for a global plant functional classification. J. Veg. Sci.

[b17] He J, Zhao X, Sun Z, Fan J, Mao S, Zhou H (2011a). Effects of the ice and snow damage to the evergreen broad-leaved forest of Jiulianshan Mountain Jiangxi Province. Guihaia.

[b18] He J, Zhao X, Zhang C, Jia Y, Fan J, Mao S (2011b). Ice and snow disasters to the evergreen broad-leaved forest in the Jiulianshan Nature Reserve in Jiangxi, China. Chin. J. Appl. Environ. Biol.

[b19] Holzmueller EJ, Gibson DJ, Suchecki PF (2012). Accelerated succession following an intense wind storm in an oak-dominated forest. Forest Ecol. Manag.

[b20] Hopkin A, Williams T, Sajan R, Pedlar J, Nielsen C (2003). Ice storm damage to eastern Ontario forests: 1998–2001. Forest. Chron.

[b21] Irland LC (2000). Ice storms and forest impacts. Sci. Total Environ.

[b22] Kollas C, Körner C, Randin CF (2014). Spring frost and growing season length co-control the cold range limits of broad-leaved trees. J. Biogeogr.

[b23] Lafon CW (2004). Ice-storm disturbance and long-term forest dynamics in the Adirondack Mountains. J. Veg. Sci.

[b24] Laurance WF, Oliveira AA, Laurance SG, Condit R, Nascimento HEM, Sanchez-Thorin AC (2004). Pervasive alteration of tree communities in undisturbed Amazonian forests. Nature.

[b25] Lewis SL, Lloyd J, Sitch S, Mitchard ETA, Laurance WF (2009). Changing ecology of tropical forests: evidence and drivers. Annu. Rev. Ecol. Evol. Syst.

[b26] Li Y, Liu X, Liao M, Yang J, Stanford CB (2009). Characteristics of a group of Hubei Golden Snub-nosed Monkeys (*Rhinopithecus roxellana hubeiensis*) before and after major snow storms. Am. J. Primatol.

[b27] Lind L, Nilsson C, Weber C (2014). Effects of ice and floods on vegetation in streams in cold regions: implications for climate change. Ecol. Evol.

[b28] Lloret F, Escudero A, Iriondo JM, Martínez-Vilalta J, Valladares F (2012). Extreme climatic events and vegetation: the role of stabilizing processes. Global Change Biol.

[b29] Luo Y, Chen HY (2013). Observations from old forests underestimate climate change effects on tree mortality. Nat. Commun.

[b30] Man X, Mi X, Ma K (2011). Effects of an ice storm on community structure of an evergreen broadleaved forest in Gutianshan National Nature Reserve, Zhejiang Province. Biodivers. Sci.

[b31] Myers N, Mittermeier RA, Mittermeier CG, Da Fonseca GAB, Kent J (2000). Biodiversity hotspots for conservation priorities. Nature.

[b32] Nadrowski K, Pietsch K, Baruffol M, Both S, Gutknecht J, Bruelheide H (2014). Tree species traits but not diversity mitigate stem breakage in a subtropical forest following a rare and extreme Ice Storm. PLoS ONE.

[b33] Peng C, Ma Z, Lei X, Zhu Q, Chen H, Wang W (2011). A drought-induced pervasive increase in tree mortality across Canada's boreal forests. Nat. Clim. Change.

[b34] R Core Team (2011). R: a language and environment for statistical computing.

[b35] Rehm EM, Lenz A, Hoch G, Körner C (2014). Spring patterns of freezing resistance and photosynthesis of two leaf phenotypes of *Hedera helix*. Basic Appl. Ecol.

[b36] Reichstein M, Bahn M, Ciais P, Frank D, Mahecha MD, Seneviratne SI (2013). Climate extremes and the carbon cycle. Nature.

[b37] Rhoads AG, Hamburg SP, Fahey TJ, Siccama TG, Hane EN, Battles J (2002). Effects of an intense ice storm on the structure of a northern hardwood forest. Can. J. Forest Res.

[b38] Shao Q, Huang L, Liu J, Kuang W, Li J (2011). Analysis of forest damage caused by the snow and ice chaos along a transect across southern China in spring 2008. J. Geogr. Sci.

[b39] Shi L, Wang H, Zhang W, Shao Q, Yang F, Ma Z (2013). Spatial response patterns of subtropical forests to a heavy ice storm: a case study in Poyang Lake Basin, southern China. Nat. Hazards.

[b40] Smith KT, Shortle WC (2003). Radial growth of hardwoods following the 1998 ice storm in New Hampshire and Maine. Can. J. Forest Res.

[b41] Steven D, Kline J, Matthiae PE (1991). Long-term changes in a Wisconsin Fagus-Acer forest in relation to glaze storm disturbance. J. Veg. Sci.

[b42] Takahashi KTK, Arii KAK, Lechowicz MJ (2007). Quantitative and qualitative effects of a severe ice storm on an old-growth beech-maple forest. Can. J. Forest Res.

[b43] Tanner EVJ, Rodriguez-Sanchez F, Healey JR, Holdaway RJ, Bellingham PJ (2014). Long-term hurricane damage effects on tropical forest tree growth and mortality. Ecology.

[b44] The Editorial Board of Flora of China (2004). Flora of China.

[b45] Walter J, Jentsch A, Beierkuhnlein C, Kreyling J (2013). Ecological stress memory and cross stress tolerance in plants in the face of climate extremes. Environ. Exp. Bot.

[b46] Weeks BC, Steven P, Hamburg HS, Vadeboncoeur MAVM (2009). Ice storm effects on the canopy structure of a northern hardwood forest after 8 years. Can. J. Forest Res.

[b47] Wright SJ, Kitajima K, Kraft NJ, Reich PB, Wright IJ, Bunker DE (2010). Functional traits and the growth-mortality trade-off in tropical trees. Ecology.

[b48] Wu Z (1980). Vegetation of China.

[b49] Xiong X, Xiong G, Xie Z (2002). The regeneration of tree species in the mixed evergreen-deciduous broad-leaved forests in the Shennongjia Mountains, Hubei Province. Acta Ecol. Sin.

[b50] Zhang M, Xiong G, Zhao C, Chen Z, Xie Z (2003). Structures and patterns of a *Fagus engleriana-Cyclobalanopsis oxyodon* community in Shennongjia area, Hubei province. Chin. J. Plant Ecol.

[b51] Zhou B, Gu L, Ding Y, Shao L, Wu Z, Yang X (2011). The great 2008 Chinese ice storm: its socioeconomic-ecological impact and sustainability lessons learned. B. Am. Meteorol. Soc.

[b52] Zhou Y, Newman C, Chen J, Xie Z, Macdonald DW (2013). Anomalous, extreme weather disrupts obligate seed dispersal mutualism: snow in a subtropical forest ecosystem. Global Change Biol.

[b53] Zhou G, Houlton BZ, Wang W, Huang W, Xiao Y, Zhang Q (2014). Substantial reorganization of China's tropical and subtropical forests: based on the permanent plots. Global Change Biol.

